# Bacterial supernatants elevate glucose-dependent insulin secretion in rat pancreatic INS-1 line and islet β-cells via PI3K/AKT signaling

**DOI:** 10.1007/s11010-018-3408-7

**Published:** 2018-07-23

**Authors:** Liza L. Ramenzoni, Richard A. Zuellig, Abbas Hussain, Roger Lehmann, Christian Heumann, Thomas Attin, Patrick R. Schmidlin

**Affiliations:** 1 Clinic of Preventive Dentistry, Periodontology and Cariology, Center of Dental Medicine, University of Zurich, Plattenstrasse 11, 8032 Zurich, Switzerland; 2 Division of Endocrinology, Diabetes and Clinical Nutrition, University Hospital Zurich, Rämistrasse 100, 8091 Zurich, Switzerland; 3 Department for Statistics, Ludwig-Maximilians-University Munich, Theresienstrasse 39/I, 80333 Munich, Germany

**Keywords:** Insulin, Gene expression, Cytokines, Microbiology, Signaling pathway

## Abstract

Diabetes and periodontitis are considered associated chronic diseases, and hyperinsulinemia in prediabetes has been shown to be present in normoglycemic animals with periodontitis. As periodontal bacterial species are significant sources of endotoxemia and may directly stimulate insulin secretion, we hypothesized that increased bacterial virulence may exert an adverse effect on rat pancreatic β-cell function via PI3K/AKT signaling. INS-1 cells and isolated pancreatic islets were cultured separately with the following supernatants: *Streptococcus anginosus, Streptococcus mutans, Fusobacterium nucleatum, Prevotella intermedia, Porphyromonas gingivalis* (*P.g*), and *Treponema denticola* (*T.d*). Supernatants were purified from single bacterial cultures and prepared at different dilutions (100 pg/ml, 50 ng/ml, 200 ng/ml, and 500 ng/ml) to challenge INS-1 and islets. Gene expression (*IL-1β, TNFα, IL-6, TLR2, TLR4, Ins1*, and *Ins2*) and insulin secretion were measured. The results showed upregulation of gene expression up to 5.5-fold, not only as a result of the different dilutions used, but also due to bacterial virulence (*p* < 0.05). *P.g* and *T.d* supernatants demonstrated an increase in insulin secretion to fivefold at hypo- and hyperglycemia, yet stimulation from hypo- to hyperglycemia stays in the same ratio. Activation of TLR4/PI3K/AKT signaling by supernatants in INS-1 cells resulted in increased *IL-1β, TNFα, IL-6* gene expression levels, and AKT phosphorylation, which were abolished by TLR4 and PI3K/AKT signaling inhibitor. We demonstrated that bacterial supernatants derived from gram-negative species increasingly stimulate insulin secretion in β-cells and TLR4 may promote inflammation by activating the PI3K/AKT signaling pathway to induce pro-inflammatory molecules. Bacterial species, depending on their virulence, appear to play a role in the relationship between periodontitis and prediabetes by promoting insulin resistance and β-cell compensatory response.

## Introduction

Periodontitis is a chronic inflammatory disease featuring an immunological host response that leads to the destruction of periodontal tissues [1]. It is considered a multifactorial cause related to several environmental, genetic and acquired risk factors and intrinsically linked to the bacterial biofilm of specific endogenous oral species [2]. A continuous inflammatory reaction of gingival tissue will lead to alveolar bone loss and breakdown of surrounding connective tissues. Oral pathogenic bacteria can produce host tissue inflammation and bacterial endotoxins/lipopolysaccharides (LPS) from affected periodontal sites are likely to invade the circulatory system and induce endotoxemia [3, 4]. Consequently, this “periodontopathogenic leakage” of bacterial products could mediate inflammatory pathologies at distant organ sites. In fact, endotoxemia has been found to affect systemic health and is an increased risk factor for pneumonia, atherosclerosis, rheumatoid arthritis, adverse pregnancy outcome, and cancer [5–8]. Additionally, bacterial endotoxins may generate molecular responses, activating inflammation reactions in organs related to insulin storage and production, culminating in insulin resistance (IR) or diabetes mellitus [9, 10].

Although some studies suggest that periodontal disease could affect the development of diabetes [11, 12] and increase the risk of diabetic complications [13, 14], little mechanistic evidence has been published on the potential impact of periodontitis on diabetes. Animal studies to date have shown that hyperinsulinemia in β-cells occurs when insulin signaling downstream of the insulin receptor is impaired in insulin target organs and bacterial endotoxins can stimulate the production of cytokines and consequently IR in insulin target organs [9]. This signaling impairment is largely due to the existence of pro-inflammatory cytokines in the blood stream [15]. In fact, it was shown that IR appears in mice with induced periodontitis by applying LPS in the gingival sulcus [16]. Data regarding the impact of the major mediators of inflammation as bacterial endotoxins on β-cell function are still not clear, as some reports revealed stimulating [17] role of LPS in β-cell insulin secretion while others [18] report inhibiting role of LPS on insulin gene expression in isolated islets. In addition, the upstream signaling pathways that activate PI3K-AKT signaling to promote inflammation response in pancreatic β-cell remain to be determined. Toll-Like Receptor 4 (TLR4) is a major component of the innate immune response and it is also present in non-immune cells as pancreatic β-cells [19–21]. Circulating bacterial endotoxins binding TLR4 have been shown to activate inflammation via differentiation factor 88 (MyD88)-independent pathways (i.e., MyD88-TRIF-PI3K/AKT pathway) and the evidence suggests that AKT1 activation in pancreatic islet cells increases β-cell mass and insulin production [22]. However, for the most part, the precise molecular and signaling mechanisms by which bacterial products affect β-cell insulin secretion are still left to explore.

As gram-negative bacterial products play an important role in the pathogenesis of periodontitis, we hypothesized that an increase in bacterial virulence may have a direct effect on INS-1 pancreatic β-cell line. To test our hypothesis, we investigated the direct effect of different bacterial pathogenicities on rat immortalized β-cell line and isolated rat pancreatic islets by measuring insulin secretion and inflammation in vitro. We further evaluated whether TLR4-mediated PI3K/AKT signaling participates in supernatant-induced pancreatic inflammation.

## Materials and methods

### INS-1 cell culture

A rat insulinoma (INS-1)-immortalized β-cell line was used to determine the effects of different bacterial supernatants on insulin production. INS-1 cells from the Division of Endocrinology, Diabetes and Clinical Nutrition, University Hospital Zurich, University of Zurich were maintained in Roswell Park Memorial Institute (RPMI) starvation or hypoglycemic medium supplemented with 10% fetal bovine serum, 100 U/ml penicillin, 100 U/ml streptomycin, 1 mM sodium pyruvate, 50 µl β-mercaptoethanol (Sigma-Aldrich, St. Louis, MO, USA) in an atmosphere of 5% CO_2_ at 37 °C in a humidified incubator. Medium was changed every 3–4 days and cells passaged once a week. The cells used in this study were between the 10th and 20th passage.

### Rat pancreatic islet isolation and culture

Six male Wistar rats weighing 250 g (no defined age) were obtained from the Division of Endocrinology, Diabetes and Clinical Nutrition, University Hospital Zurich, University of Zurich. The animals were kept at 23 °C under a 12-h light–dark cycle, with free access to food and water. All experiments were conducted in accordance with the Swiss Animal Protection Laws and procedures were conducted with the relevant approval of the appropriate authorities. Islets were harvested from pancreata of male rats by collagenase (collagenase NB8, Serva) digestion using the method of pancreas digestion, as previously described [23]. Islets were picked under a dissecting microscope, rinsed three times in Hanks solution, and cultured overnight in an incubator under 5% CO_2_. The RPMI culture medium used contained 5.5 mM d-glucose (Invitrogen, Carlsbad, CA), 10% FCS (fetal bovine serum) (HyClone Laboratories, Inc., Logan, UT), 100 U/ml penicillin, 100 U/ml streptomycin, and 40 µg/ml gentamicin (Invitrogen, Carlsbad, CA). Islets suspension was cultured in a 40-µm cell strainer (Falcon, Corning, NY, USA), and medium was isolated after glucose stimulation to determine insulin secretion.

### Bacterial strains and cultured supernatants

Six bacterial species were used in this study: *Streptococcus mutans* UAB159 (OMZ 918), *Streptococcus anginosus* ATCC9895T (OMZ 871), *Prevotella intermedia* ATCC25611T (OMZ 278), *Fusobacterium nucleatum* KP-F8 (OMZ 598), *Porphyromonas gingivalis* ATCC33277T (OMZ 308), and *Treponema denticola* ATCC35405T (OMZ 661). *T. denticola* (*T.d*) was cultivated anaerobically in 10 ml of spirochetes medium (OMIZ-W68) at 37 °C. The other strains were cultured on Columbia Blood Agar (CBA) plates under the same anaerobic conditions. Liquid pre-cultures were prepared by inoculation of the bacterial colonies from the CBA plates into a modified fluid universal medium. The growth was obtained after 12–18 h of incubation in a shaking incubator at 37 °C. All bacterial cultures were adjusted to OD_550 nm_ = 1.0 and centrifuged at 600×*g* for 10 min at 4 °C. The supernatants were finally filter-sterilized (0.2 µm filter) and made free from bacterial debris/cells and stored at − 80 °C. Then, supernatants were added to distinct working solutions in INS-1 cell culture media and made up to final dilution of 100 pg/ml, 50 ng/ml, 200 ng/ml, and 500 ng/ml as applied in previous studies [24–26] for comparison among each bacterial species (i.e., 100 pg/ml means 1:10^10^ dilution). Cell wall fragments, extracellular proteins, and secreted bacterial products typically compose the content of bacterial supernatant, but were not specifically identified or quantified for the series of experiments in this study. For the mixed supernatants, the dilution to their final concentrations (100 pg/ml, 50 ng/ml, 200 ng/ml, and 500 ng/ml) was performed as follows: each of the six bacterial species was partially diluted in order to achieve the final dilution with all bacterial species mixed together (i.e., 16.67 pg/ml dilution for each of the six bacterial species, when all species mixed resulted in 100 pg/ml).

### Glucose-stimulation insulin secretion and insulin measurement in INS-1 cells and isolated rat pancreatic islets

In order to validate their glucose response, INS-1 cells were first incubated in serum-free culture medium (supplements described above) containing 3.3 mM glucose at 37 °C. After 24 h, the medium was replenished with RPMI, supplements and with 3.3 mM glucose. The bacterial supernatants of *P. gingivalis* (*P.g*) and *T.d* and mixed supernatants were added to working solutions/media and made up to a final dilution (0, 100 pg/ml, 50 ng/ml, 200 ng/ml, and 500 ng/ml) in single 24-well plates specific for each bacterial strain overnight (16 h). Cells were washed and incubated with Krebs–Ringer bicarbonate HEPES buffer (KRBH, 135 mM NaCl, 3.6 mM KCl, 5 mM NaHCO_3_, 0.5 mM NaH_2_PO_4_, 0.5 mM MgCl_2_, 1.5 mM CaCl_2_, and 10 mM HEPES, pH 7.4, and 0.1% bovine serum albumin) (Sigma, St. Louis, MO), 3.3 mM glucose (hypoglycemic glucose concentration) and supernatants in different dilutions for 1 h at 37 °C. This minimal glucose solution was removed and replaced with KRBH plus 16.7 mM glucose (hyperglycemic glucose concentration) with the same supernatant dilutions, and the cells were reincubated for 1 h. For the insulin measurement in isolated rat pancreatic islets, 40 islets/well of 150–200 µm in diameter kept in 24-well plates were also pre-incubated with RPMI and supplements overnight with bacterial supernatants of *P.g, T.d* and mixed supernatants (0, 100 pg/ml, 50 ng/ml, 200 ng/ml, and 500 ng/ml) in single 24-well plate specific for each bacterium. The pre-incubation medium was then replaced with KRBH buffer supplemented with different glucose concentrations of 3.3 mM glucose and supernatants for 1 h and subsequently stimulated for 1 h with 16.7 mM glucose with supernatants. At the end of each stimulation for INS-1 and isolated islets, the medium was collected, cleared by centrifugation, and stored at − 80 °C for later analysis. Supernatants were assayed for insulin contents using Mercodia Ultra sensitive rat insulin ELISA kit (Crystal Chem, IL, USA). Secreted insulin levels were presented as % content.

### Quantitative real-time PCR (RT-qPCR)

Gene expression analysis was conducted using only INS-1 cell line which was seeded with hypoglycemic medium and then treated with isolated bacterial supernatants and mixed supernatants (0, 100 pg/ml, 50 ng/ml, 200 ng/ml, and 500 ng/ml) for 16 h. Briefly, total RNA was extracted from the cells with Trizol Reagent (Gibco, Life Technologies, Carlsbad, CA, USA) and quantified using NanoDrop ND-1000 (Thermo-Fisher Scientific, Wohlen, Switzerland). cDNA was synthesized using an iScript kit (Bio-Rad, Hercules, CA, USA). RT-qPCR reactions were carried out on a CFX96 real-time PCR system (Bio-Rad) by incubating initially for 2 min at 50 °C and 10 min at 95 °C, followed by 40 cycles of 15 s at 95 °C and 1 min at 60 °C and run in a total reaction volume of 15µl, containing 7.5µl of SYBR®Green PCR Master Mix (Life Technologies, Zug, Switzerland), 6 µl of sample (1 ng), and 1.5 µl of primer solution of 1 µM (mixture of forward and reverse primers). The authors selected specific genes related to two pathways for this study: (1) rat immune response: *TLR2, TLR4, TNFα, IL-6*, and *IL-1β* and (2) rat insulin signaling: *Ins1, Ins2*. The following PCR primers were used in three independent experiments for further specific analysis: *TLR2*, 5′-GTACGCAGTGAGTGGTGCAAGT-3′ and 5′-GGCCGCGTCATTGTTCTC-3′, *TLR4*, 5′-AATCCCTGCATAGAGGTACTTCCTAAT-3′ and 5′-CTCAGATCTAGGTTCTTGGTTGAATAAG-3′, *TNF-α*, 5′-CCAGGAGAAAGTCAGCCTCCT-3′ and 5′-TCATACCAGGGCTTGAGCTCA-3′, *IL-1β*, 5′-CACCTCTCAAGCAGAGCACAG-3′ and 5′-GGGTTCCATGGTGAAGTCAAC-3′, *IL-6*, 5′-CGAAAGTCAACTCCATCTGCC-3′ and 5′-GGCAACTGGCTGGAAGTCTCT-3′, *Ins1*, 5′-TCTTCTACACACCCAAGTCCCG-3′ and 5′-AGTGCCAAGGTCTGAAGATCCC-3′, *Ins2*, 5′-ATCCTCTGGGAGCCCCGC-3′ and 5′-AGAGAGCTTCCACCAAG-3′, *GAPDH*, 5′-GCTCTCTGCTCCTCCCTGTT-3′ and 5′-CACACCGACCTTCACCATCT-3′. From the *C*_q_ values obtained with the qPCR, the expression levels of transcripts were calculated by using the comparative *C*_t_ method (${2^{ - \Updelta \Updelta {C_{\text{T}}}}}$ formula) after normalization to the internal reference gene (*GAPDH*). Results are presented in means ± standard deviations (SDs).

### TLR4-mediated PI3K/AKT signaling pathway inhibition

INS-1 cells were cultured (2 × 10^6^ cells /well) to assess the production of cytokines after stimulation with mixture of supernatants (500 ng/ml) and TLR4, PI3K, and AKT inhibitors, each inhibitor in separate experiments for 24 h. The blocking experiment was performed with TLR4 inhibitor (500 nM viral inhibitory peptide, VIPER), PI3K inhibitor (500 nM NVP-BEZ235) and Pan AKT inhibitor (450 nM MK2206) (Imgenex, USA), in order to examine any change in the expression of cytokines *IL-1β, IL-6, TNF-α* (RT-qPCR) and AKT protein (western blot). After 24 h of exposure, total RNA or total protein content was collected in triplicate, pooled, and frozen at − 80 °C until testing.

### Western blot analysis

Western blotting was performed to determine the protein expression of AKT after inhibition of TLR4, PI3K, and AKT. Protein was extracted with radio-immunoprecipitation assay (RIPA) buffer containing a protease inhibitor cocktail and centrifuged at 12,000× *g* for 15 min at 4 °C. The supernatant protein was quantified by bicinchoninic acid assay (BCA, Thermo Fisher Scientific, Rockford, USA) and stored at − 80 °C. Total lysates were resolved in SDS-PAGE. Proteins were blotted onto a nitrocellulose membrane and incubated with primary antibodies and the corresponding secondary antibodies. Immune complexes were visualized by the use of an enhanced chemiluminescence western blotting system (Bio-Rad, Richmond, CA). Antibodies used for immunoblotting were as follows: mouse monoclonal antibody against pan AKT (40D4) and mouse monoclonal antibody against GAPDH (Cell Signaling Technology, Beverly, MA, USA).

### Statistical analysis

**Fig. 1:**
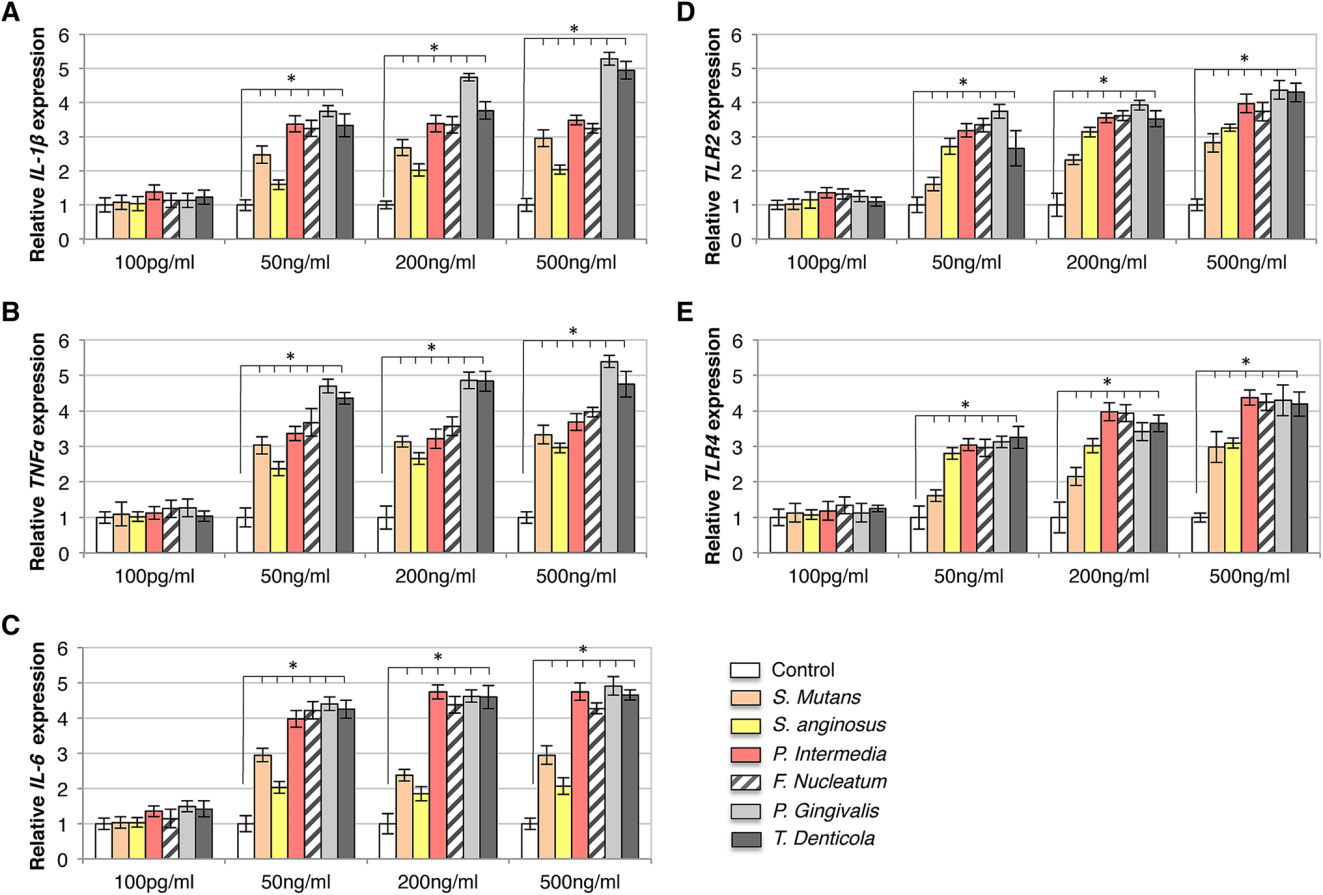
Bacterial supernatant treatments stimulated upregulation of immune response gene expressions in INS-1 β-cells. Quantitative PCR analysis shows increased expression of *IL-1β* (**a**), *TNFα* (**b**), *IL-6* (**c**), *TLR2* (**d**), *and TLR4* (**e**) under exposure to supernatants (*S. anginosus, S. mutans, F. nucleatum, P. intermedia, P. gingivalis*, and *T. denticola*) compared to control (white bars). Upregulation was found to be higher when exposed to *P. gingivalis* and *T. denticola* and with 50, 200, and 500 ng/ml of supernatants. *x*-axis: bacterial species and concentration of bacterial supernatants, *y*-axis: relative gene expressions. Data are mean ± S.D. of at least three independent experiments; *p < 0.05

**Fig. 2:**
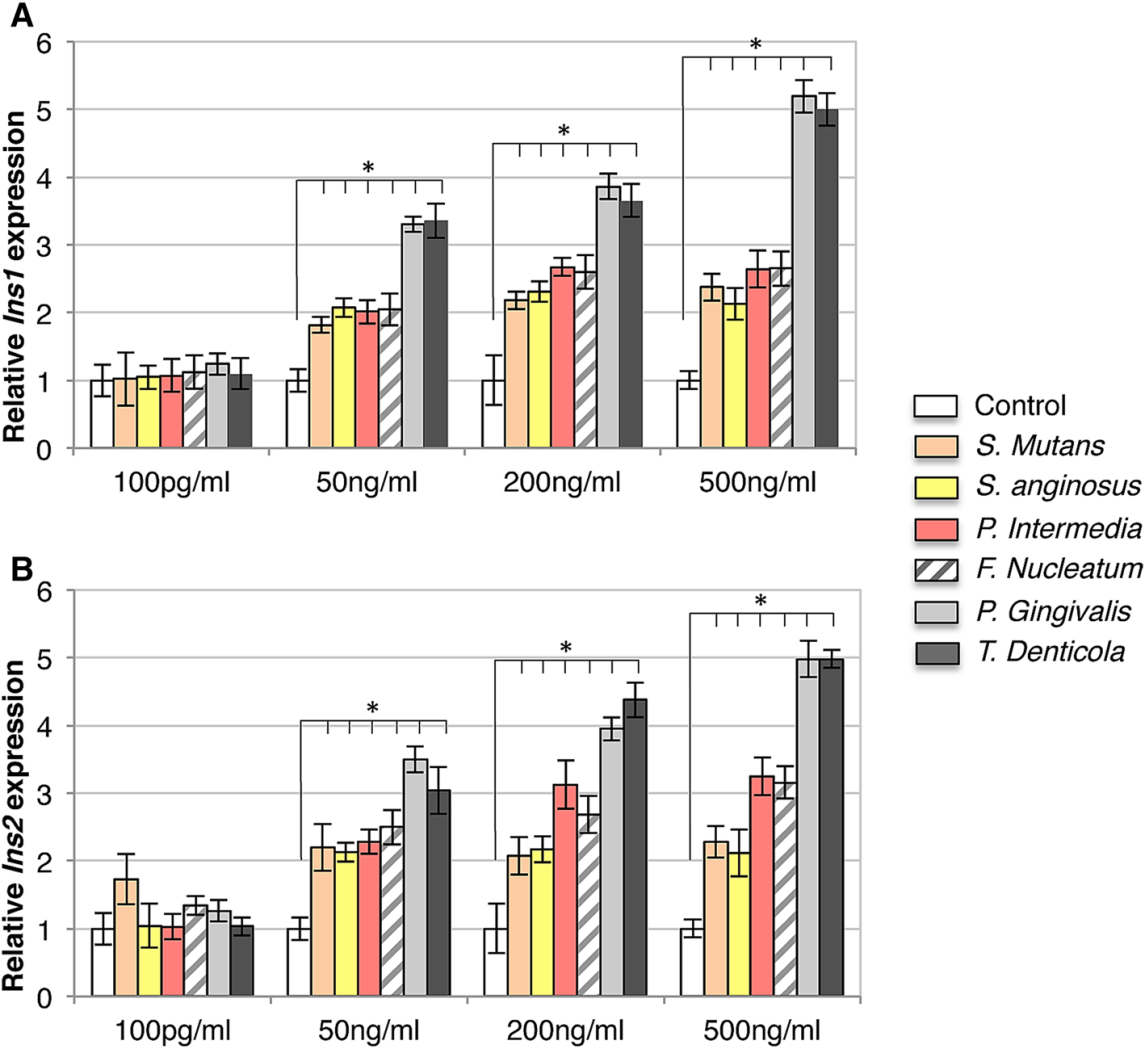
Bacterial supernatant treatments stimulated upregulation of insulin gene expressions in INS-1 β-cells. Increased expression of *Ins1* (**a**) and *Ins2* (**b**) genes was found in 50, 200, and 500 ng/ml compared to control (white bars). The supernatants of *P. gingivalis* and *T. denticola* showed a prominent increase in gene expression compared to other bacterial species. *x*-axis: bacterial species and concentration of bacterial supernatants, *y*-axis: relative genes expression. *p < 0.05. Mean ± SD

**Fig. 3:**
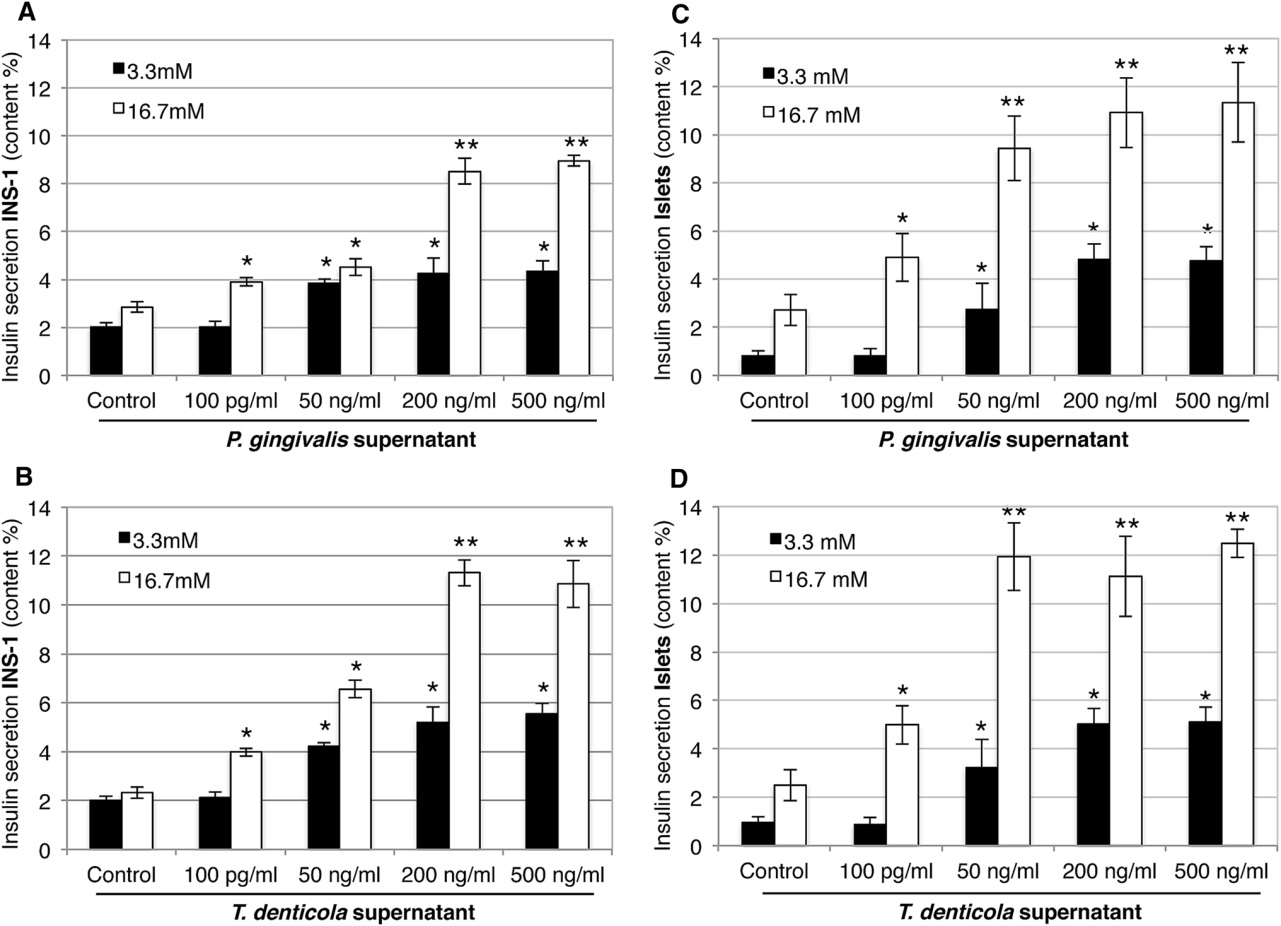
Effect of bacterial supernatant treatments of *P. gingivalis* and *T. denticola* on insulin secretion in INS-1 β-cells (**a, b**) and pancreatic islets (**c, d**) stimulated with 3.3 mM glucose (black bars) and 16.7 mM glucose (white bars). The glucose-stimulated insulin secretion was analyzed by ELISA and insulin secretion was presented as % content, showing similarly increased levels of secreted insulin in INS-1 β-cells and islets infected with *P. gingivalis* and *T. denticola* supernatants (50, 200, and 500 ng/ml) in stimulations with both 3.3 and 16.7 mM glucose. *x*-axis: bacterial species and concentration of bacterial supernatants, *y*-axis: % content in insulin secretion in INS-1 β-cells and islets. Data are mean ± SD of at least three independent experiments; *p < 0.05, **p < 0.01

**Fig. 4:**
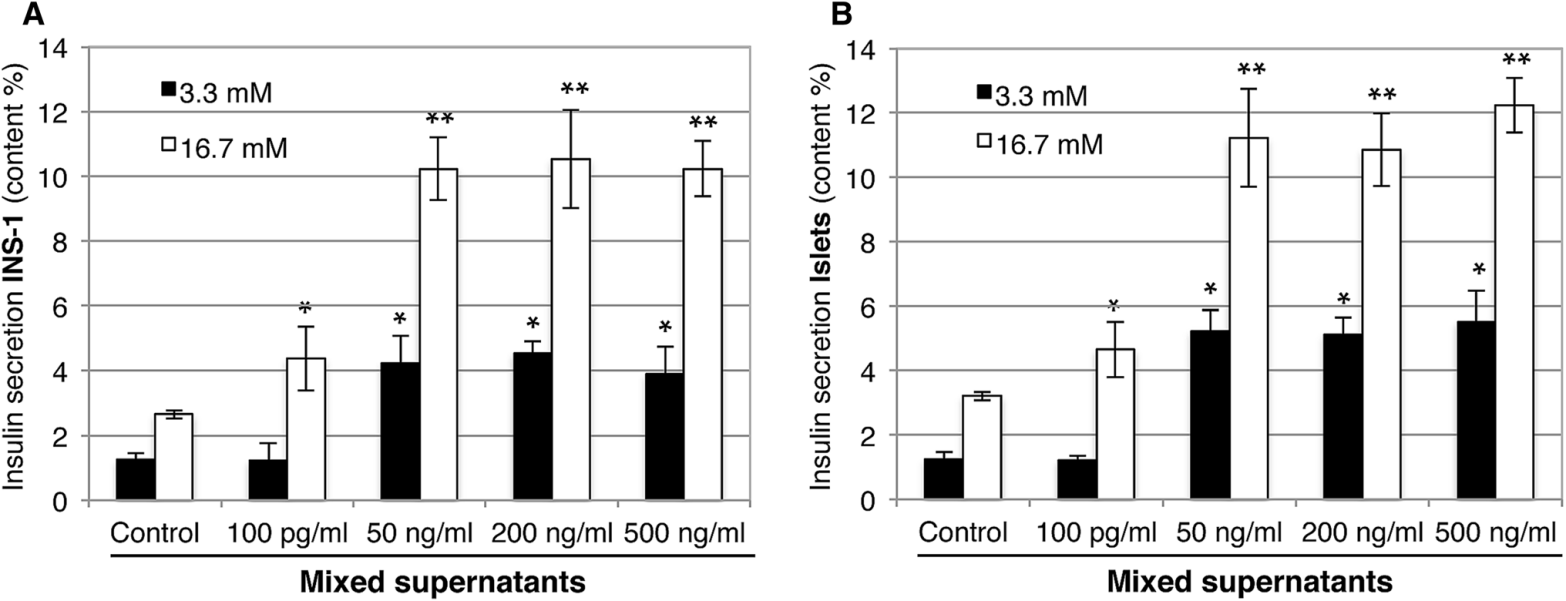
Effect of mixed bacterial supernatants on insulin secretion in INS-1 β-cells (**a**) and pancreatic islets (**b**) stimulated with 3.3 mM glucose (black bars) and 16.7 mM glucose (white bars). The glucose-stimulated insulin secretion (ELISA) upon mixed supernatant treatment showed higher increases for INS-1 and pancreatic islets in 50, 200, and 500 ng/ml compared with the control. Data are mean ± SD of at least three independent experiments;*p < 0.05, **p < 0.01

**Fig. 5:**
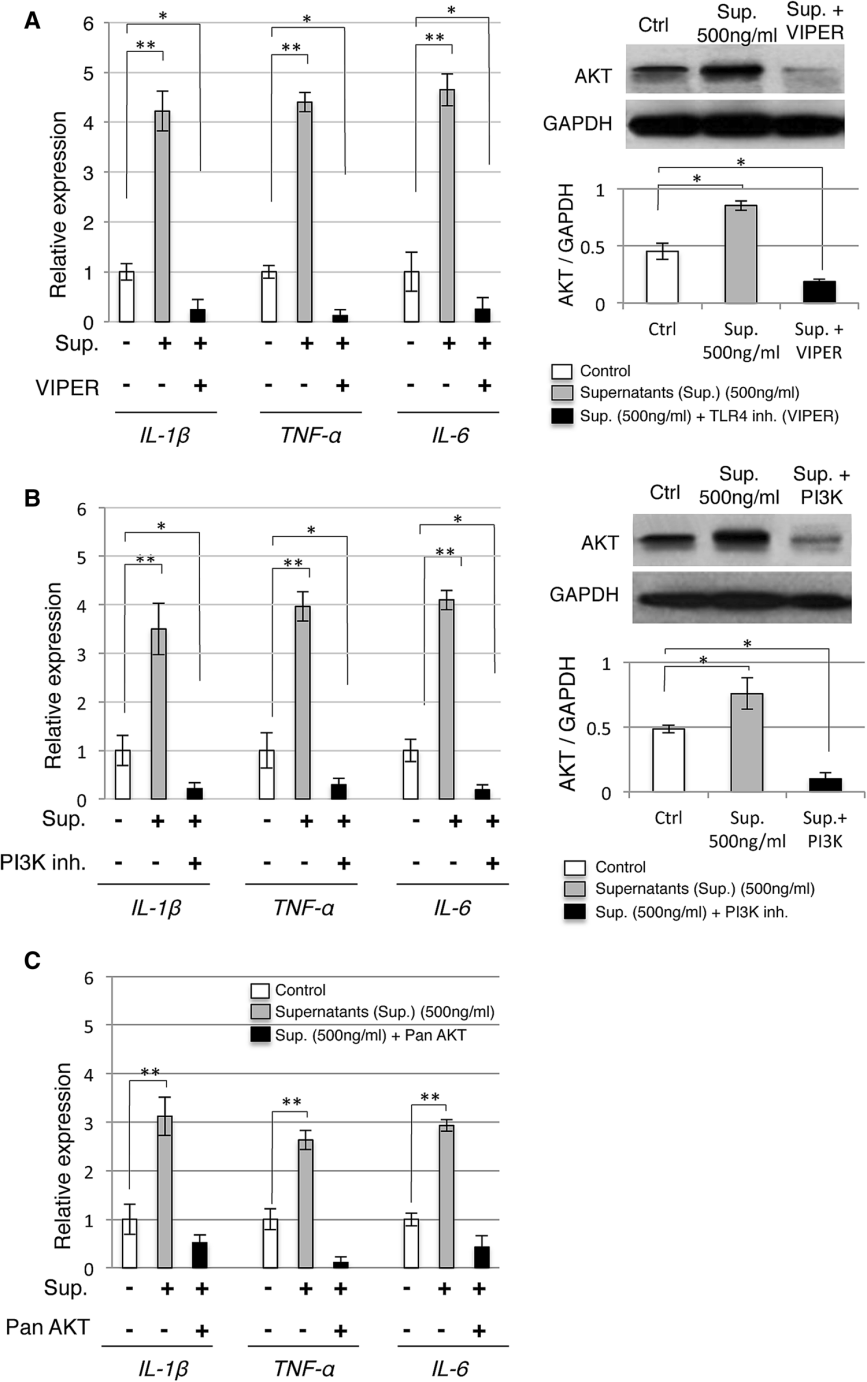
Mixed bacterial supernatants trigger TLR4-mediated PI3K/AKT signaling in INS-1. For analysis of mRNA expression of *IL-1β, TNFα, IL-6*, INS-1 cells were cultivated in the presence or absence of mixed supernatants (500 ng/ml), with or w/o TLR4/VIPER (**a**), PI3K inhibitor (**b**) and Pan AKT inhibitor (**c**) for 24 h. Western blot was performed to detect activation state of AKT and its concentration activation was determined (**a, b**). Relative mRNA expression was determined by RT-PCR. Molecular weights of bands are expressed in kDa. Representative immunoblots are shown. Graphs are presented as means ± SEM from at least three independent experiments. Statistically significant difference in comparison to control, INS-1 in the absence of supernatants (set as 1): by *t*-test: *p < 0.05

Statistical analysis was completed using IBM SPSS software (IBM SPSS Statistics for Windows, version 23.0; IBM Corp., Armonk, NY). Linear mixed model statistics were performed to simultaneously explore the effect of the different bacterial supernatants, the different dilutions and the genes examined. The group differences were identified using post hoc tests with a Bonferroni correction for multiple comparisons. We assume that the repeated measurements taken from the same bacterial supernatant with the same dilution are correlated. Simultaneous models usually increase the power of the statistical analysis compared to a large series of pairwise comparisons. In Figs. 1, 2, 3, 4, and 5, the data were presented together with the significance of paired tests between the different configurations of genes, bacterial supernatants, and dilution. The quantitative data are presented as mean $\pm$SD; *p* ≤ 0.05 was considered to be statistically significant.

## Results

### Different supernatant dilutions upregulated expression of immune response and insulin genes in INS-1 β-cells in hypoglycemic condition

The qPCR analysis showed an upregulation of *IL-1β, IL-6, TNF-α, TLR2*, and *TLR4* genes in INS-1 β-cells after exposure to different dilutions (100 pg/ml, 50 ng/ml, 200 ng/ml, and 500 ng/ml) of the bacterial supernatants, as compared to the INS-1 β-cell control treatment (Fig. 1). Upregulation was found to be higher in β-cells when exposed to supernatants of *P.g* and *T.d*, both of which are well known and powerful periodontal pathogens. Thus, the increase of gene expression was dependent on the supernatant dilution and the increase in bacterial virulence: expression was up to 5.5-fold higher in the INS-1 cells exposed to higher concentrations of supernatants (i.e., 50–500 ng/ml) and for INS-1 cells in supernatants derived from pathogenic gram-negative bacteria. The statistical linear mixed-model analysis confirmed the explorative and pairwise-comparison results.

The statistical model I included the *IL-1β, IL-6, TNF-α, TLR2*, and *TLR4* genes, the different dilutions, and the bacterial supernatants as fixed-effect factors. All main factors were significant (*p* < 0.05). Two interactions (Bacteria * Dilution and Bacteria * Gene) were also significant (*p* < 0.05).

Supernatants also increased the expression of the two insulin-signaling pathway genes, *Ins1* (a) and *Ins2* (b), by 2- to fivefold (*p* < 0.05) (see Fig. 2). The change in gene expression of *Ins1* and *Ins2* was found to be highest when cells were exposed to the bacterial supernatant of *P.g* and *T.d*.

The statistical model II included *Ins1* and *Ins2*, different dilutions, and the bacterial supernatants as fixed-effect factors (*p* < 0.05). All main factors were significant (*p* < 0.05). One interaction (Bacteria * Dilution) was also significant (*p* < 0.05).

In a separate experiment, a mix of all bacterial supernatants at identical final dilutions was used to test the change in gene expression in INS-1 cells. The results showed an increase in gene expression (fivefold) for a dilution of 100 pg/ml and were significant as compared to the expression in single bacterial species supernatant stimulation (Table 1), which provided no evidence of change.

**Table 1:** Change in gene expression subsequent to the incubation with mixed bacterial supernatants in different dilutions

Genes	100 pg/ml		50 ng/ml		200 ng/ml		500 ng/ml	
	Fold change	*p*-values	Fold change	*p*-values	Fold change	*p*-values	Fold change	*p*-values
*IL-1β*	+ 4.51	0.028	+ 4.72	0.021	+ 5.37	0.021	+ 5.44	0.028
*IL-6*	+ 4.75	0.029	+ 4.94	0.015	+ 5.59	0.026	+ 5.65	0.022
*TNFα*	+ 4.99	0.015	+ 4.99	0.043	+ 5.53	0.017	+ 5.86	0.023
*TLR2*	+ 4.90	0.005	+ 5.15	0.028	+ 5.61	0.026	+ 5.51	0.015
*TLR4*	+ 5.07	0.002	+ 5.20	0.026	+ 5.78	0.031	+ 5.87	0.019
*Ins1*	+ 4.28	0.041	+ 4.50	0.013	+ 5.18	0.038	+ 5.37	0.012
*Ins2*	+ 4.30	0.032	+ 4.60	0.025	+ 5.31	0.041	+ 5.31	0.025

### Treatment with supernatant dilutions of *P.g* and *T.d* elevated glucose-stimulated insulin secretion in INS-1 cells and isolated islets in both hypo- and hyperglycemic conditions

As *P.g* and *T.d* species caused higher gene expression, the measurement of the β-cell insulin secretion in INS-1 and islets was only performed using these two bacterial species. Figure 3 depicts the statistically significant (*p* < 0.05) increase in insulin production found after treatment in INS-1 cells (a, b) and islets (c, d) in hypoglycemic (3.3 mM) and hyperglycemic (16.7 mM) conditions. In low glucose (3.3 mM) for INS-1 and islets, the application of a 50 ng/ml supernatant increased the insulin secretion up to threefold, while higher concentrations (200, 500 ng/ml) induced a maximum of a $\approx$ fivefold increase in secretion. The insulin values for high (16.7 mM) glucose were approximately 4.2–5-fold compared to those of low (3.3 mM) glucose. Nevertheless, the stimulation index stays the same at 2.5-fold for both conditions, which is a normal stimulation index of insulin secretion in those conditions. A 100 pg/ml supernatant dilution did not lead to any significant change in insulin secretion for INS-1 and islets in low-glucose condition.

The statistical model III included the two bacterial supernatants (*P.g* and *T.d*) with different dilutions as factors for INS-1 cells and islets in the low-/high-glucose conditions. All factors and the Bacteria * Dilution interaction were significant (*p* < 0.05).

Insulin secretion under the treatment with mixed supernatants was 5.5-fold higher in low (3.3 mM) glucose conditions and 5× higher in high (16.7 mM) glucose conditions for both INS-1 cells (a) and islets (b) with the application of 50, 200, and 500 ng/ml of supernatants, whereas high (16.7 mM) glucose in the same supernatant dilutions induced a $\approx$ 2.5-fold increase.

### Inhibition of TLR4/PI3K/AKT signaling down-regulates INS-1 cell cytokine production during bacterial mixed supernatant stimulation

To further examine the expression profiles of *IL-1β, IL-6, TNF-α*, RT-qPCR was adopted to detect inflammation after inhibition of TLR4/PI3K/AKT signaling and mixed supernatant treatment. VIPER was found to inhibit supernatant-induced *IL-1β, IL-6, TNF-α* production compared with the control group (*p* < 0.05) (Fig. 5a). The results of western blotting confirmed decrease in AKT protein production in the presence of supernatants and VIPER, which is in accordance with the results of RT-qPCR (Fig. 5a). Similar RT-qPCR results were found by inhibiting PI3K (Fig. 5b) and AKT signaling (Fig. 5c), where *IL-1β, IL-6, TNF-α* gene expression was also upregulated with supernatant treatment whereas downregulated with the inhibitor and supernatant treatment. Taken together, the patterns of TLR4 expression were significantly associated with the presence of supernatant in the medium, suggesting that TLR4 activation may result in upregulation of PI3K/AKT signaling.

## Discussion

The purpose of this study was to ascertain the importance of the bacterial virulence and its direct effect on pancreatic β-cell insulin secretion and inflammation response. Findings suggest that bacterial supernatants, especially those derived from virulent gram-negative bacteria (*P.g* and *T.d*) in higher concentrations are able to augment insulin secretion in β-cells either in hypo- or hyperglycemia. The latter result contradicts previous studies evaluating the effect of purified LPS on β-cells in hyperglycemia; in those studies, no significant change in insulin secretion was detected [17]. The selection of the supernatant dilution for our study was based on previous in vivo and in vitro assessments that have also investigated different concentrations of LPS (0–500 ng/ml) [17, 24, 25]. These studies indicated that the elevation in LPS concentration may play a key role in low-grade systemic inflammation, IR, and type-2 diabetes [26–28]. A number of cytokine genes are linked to diabetes with an inflammation-related etiology and induced immune response on β-cells [29]. Cytokines play an important role in the procedure of β-cell failure and IL-1β; TNF-α and IL-6 have been shown to diversely regulate pancreatic β-cell function leading to inflammation [29] in prediabetes patients [32, 33]. In fact, pro-inflammatory factors that are present at high levels in the blood of patients with type-2 diabetes are IL-1 dependent, and blocking IL-1 activity has been shown to reduce their concentrations [30]. Similarly, elevated levels of circulating IL-6 can predict the incidence of type-2 diabetes in predisposed individuals [31]. Additionally, TLR2 and TLR4 activation by LPS also triggers the secretion of other pro-inflammatory cytokines that contribute to IR [32]. In this study, the expression pattern of the genes *IL-1β, IL-6, TNF-α, TLR2, TLR4, Ins1*, and *Ins2* showed upregulation under exposure to 50, 200, and 500 ng/ml dilutions of supernatants from different bacteria. For this gene expression analysis, the INS-1 cells were not under any metabolic stress (low glucose level) and bacterial supernatants appear to have a direct influence on the INS-1 β-cells. Thus, the increase of gene expression was most likely dependent on the supernatant dilution and the increase in bacterial virulence: expression was higher in the INS-1 cells exposed to higher concentrations of supernatants (i.e., 50–500 ng/ml) and of supernatants derived from pathogenic gram-negative bacteria (*P.g* and *T.d*; Figs. 1, 2). As the change in gene expression of *Ins1* (Fig. 2a) and *Ins2* (Fig. 2b) was found to be higher after exposure to the bacterial supernatant of *P.g* and *T.d*, the glucose-stimulated insulin secretion was conducted using only those two bacterial species for INS-1 and isolated pancreatic islets. Treatment with supernatants elevated glucose-stimulated insulin secretion in INS-1 cells and isolated islets in both hypo- and hyperglycemic conditions (Fig. 3). Concentration of 100 pg/ml supernatant concentration was too low to induce any change in insulin secretion in low-glucose condition (Fig. 4). Additionally, the treatment with mixed supernatants showed upregulation for all dilutions (Table 1), which was not observed with the use of single species alone (Fig. 1) and elevated glucose-stimulated insulin secretion in INS-1 cells and isolated islets. According to current understanding, periodontitis is mainly caused by a pathogenic subgingival biofilm [1, 2] and not by single bacterial species. And the diversity in the species can shape development, function, structure, and virulence of the biofilm [33]. Throughout the progression of periodontitis, the biofilm releases several different bacterial by-products into the crevicular environment that, depending on the virulence of their content, may effectively stimulate inflammation [1, 4]. Those virulence factors may boost the bacterial capacity for infection. For instance, as opposed to LPS, the outer membrane vesicles stimulate maturation and release of cytokines by macrophages and dendritic cells and may promote the pathogenesis of infections [34–36]. Each of the gram-negative species used in the supernatant mixture possesses a large number of virulence factors relevant to inducing inflammation. The authors believe that the mixture of these virulence factors could have a synergy and consequently aggravate the pancreatic cell response. In comparison with the literature, polymicrobial synergy due to combined virulence factors can occur when infections are caused by more than one species and they are more severe than the sum of the individual species acting alone [37].

Moreover, we uniquely reported the requirement for TLR4 and PI3K/AKT signaling in bacterial supernatant accretion of β-cell inflammation gene expression. This increase was observed in previous studies that demonstrated the influence of purified LPS on pancreatic cells [26, 32] and identified that LPS may cause an increase in insulin production at normoglycemic levels [17]. Secretion of *IL-1β, IL-6, TNF-α* was highly inducible by supernatant treatment alone, whereas ATK/PI3K inhibition decreased *IL-1β, IL-6, TNF-α* release after supernatant treatment (Fig. 5). A direct effect of the supernatant challenge on TLR4 signaling appeared to significantly contribute to the supernatant-induced β-cell inflammation through the ATK/PI3K signaling. Accordingly, it was also reported that TLR4 activation by endotoxins resulted in enhanced AKT phosphorylation while silencing TLR4 with siRNA resulted in the opposite effect, indicating activation through PI3K/AKT signaling pathway [38]. It can be speculated that other bacterial by-products in the supernatants, not just LPS, influenced our results and led to increased insulin secretion under high-glucose condition for INS-1 cells and isolated pancreatic islets. However, it is unclear if increased insulin secretion occurred in response to impaired insulin signaling on insulin target organs in vivo, resulting in β-cell compensation, or if other factors stimulated insulin secretion. Insights into the mechanism of hyperinsulinemia may be key to understanding how periodontal bacteria influence the development of diabetes and underline the need for further investigation.

This study has taken a step in the direction of defining the direct impact of the type of periodontal microbiota on insulin regulation by activating the TLR4-mediated PI3K/AKT signaling pathway to promote inflammation. Our data demonstrated to some extend that gram-negative bacterial supernatants induce cytokine expression and increase insulin production. To our knowledge, we are the first to report the effect of whole bacterial supernatants on pancreatic cells. This study certainly cannot be regarded as conclusive since it has not been shown whether bacterial products could directly reach pancreatic cells in a living organ even though clinical studies reported short-time bacteremia in the peripheral blood of periodontal patients [3, 4]. Since sufficient evidence has not been reported, the proof such as isolation of bacterial products from the pancreas of diabetic animals using an experimental periodontitis model with bacteria would ideally still be needed. In addition, there is no strong evidence for direct impact of the type of periodontal microbiota on diabetic or glycemic regulation.

In conclusion, we have demonstrated that supernatants of different bacteria in various dilution rates induce pro-inflammatory cytokines and increase insulin secretion in the pancreatic β-cell line INS-1 and isolated pancreatic islets in low and high glucose through the TLR4-mediated PI3K/AKT signaling. The findings come in direct support to sustain oral bacterial supernatants as significant contributors for the development of β-cell inflammation and compensation, which may contribute to prediabetes in individuals with periodontitis.
